# Mindfulness and compassion training on daily work with patients and within the multiprofessional palliative care team: a retrospective self-assessment study

**DOI:** 10.1186/s12904-023-01158-9

**Published:** 2023-04-10

**Authors:** Franziska Lautwein, Manuela Schallenburger, Alexandra Scherg, Daniel Schlieper, André Karger, Yesche Udo Regel, Jacqueline Schwartz, Martin Neukirchen

**Affiliations:** 1 Interdisciplinary Centre for Palliative Medicine, Medical Faculty, Heinrich Heine University Düsseldorf, Moorenstr 5, 40225 Düsseldorf, Germany; 2 Cusanus Krankenhaus, Karl-Binz-Weg 12, 54470 Bernkastel-Kues, Germany; 3 Klinikum Links Der Weser, Senator-Weßling-Straße 1, 28277 Bremen, Germany; 4 Clinical Institute of Psychosomatic Medicine and Psychotherapy, Medical Faculty, Heinrich Heine University Düsseldorf, Moorenstr 5, 40225 Düsseldorf, Germany; 5 Paramita, Bonn, Germany; 6 Department of Anesthesiology, Medical Faculty, Heinrich Heine University Düsseldorf, Moorenstr. 5, 40225 Düsseldorf, Germany

**Keywords:** Skills and attitude, Mindfulness, Compassion, Palliative care, Self-care, Resilience, Team building

## Abstract

**Background:**

Palliative care teams work under challenging conditions in a sensitive setting with difficult tasks. The multi-professional team can play an important role. Mindfulness and compassion-based practices are used to build resilience. Our aim was to examine (1) feasibility and acceptability, (2) satisfaction and impact, and (3) opportunities and limitations of a mindfulness course.

**Methods:**

An eight-week mindfulness and compassion course was delivered in a university-based specialized palliative care unit. A meditation teacher provided preparatory evening sessions and meditation exercises that could be integrated into daily activities. The scientific analysis of the course was based on a questionnaire developed for quality assessmentThe first two parts consisted of demographic, Likert-type, and free-text items. Part 3 consisted of learning objectives that were self-assessed after finishing the course (post-then). In the analysis, we used descriptive statistics, qualitative content analysis, and comparative self-assessment.

**Results:**

Twenty four employees participated. 58% of participants attended 4 or more of the 7 voluntary mindfulness days. 91% expressed moderate to high satisfaction and would recommend the palliative care program to others. Three main categories emerged in the qualitative content analysis: providing feedback on the course, personal impact, and impact on professional life. The opportunity for self-care in a professional context was highlighted. Learning gains (CSA Gain) were high (38.5–49.4%) in terms of knowledge and techniques, moderate (26.2–34.5%) in terms of implementation of learned skills, and rather low (12.7–24.6%) in terms of changes to attitude.

**Conclusion:**

Our evaluation shows that the participants of a mindfulness and compassion course considered it as a feasible and welcome tool to familiarize a multi-professional palliative care team with self-care techniques.

**Trial registration:**

Internal Clinical Trial Register of the Medical Faculty, Heinrich Heine University Düsseldorf, No. 2018074763 (registered retrospectively on 30^th^ July 2018).

**Supplementary Information:**

The online version contains supplementary material available at 10.1186/s12904-023-01158-9.

## Background

The work of palliative care teams is especially challenging in that they work with seriously ill and dying patients as well as their highly stressed families. Furthermore, they are also confronted with stressors like work overload, understaffing or lack of organization. Despite these challenges, most palliative care team members do not perceive end of life care as a heavy burden, but see their work as a source of personal and professional fulfilment and meaningfulness [1–3]. Professional fulfilment and meaningfulness are protective factors against stressors and help to maintain a certain resilience [4, 5]. According to a nationwide survey in Germany, besides one´s own family and private life, as well as aspects like humour and compassion, the most important protective factor is the team itself [6]. At the same time, teamwork is directly affected by stressors or appears to be a potential stressor itself concerning aspects like irritability, tension between the multi-professional groups or emotional retreat [6].

Interprofessional teamwork is based on the collaboration of different people with different backgrounds, expertise and experience. This can lead to challenges such as different perspectives, poor communication or role conflicts. Nevertheless, collaboration focuses on the exchange of different knowledge and skills, and can be based on three points: diversity, friction and harmonization. Diversity means the inclusion of different professional perspectives. While friction means the overlapping of ideas with possible divergent interest, harmonization brings these ideas together into a common force. The combination of those three points can result in effective team collaboration, both for the team and for the patient care [7]. Cooperation in a multi-professional team can be both, a potential stressor and an important preventive factor. Therefore it has a high relevance for the health of the employees and thus, ultimately for good and sustainable patient care. Against this background, an investment in strengthening resilience as well as compassion and empathy of employees seems essential.

In this regard, techniques from the field of mind–body-medicine such as mindfulness and compassion are feasible instruments [8, 9]. As their suitability, especially concerning stress-related illnesses and empathic stress has been shown in various studies [10, 11], they appear to be a promising and still little explored option in the field of palliative care [12, 13].

### Mindfulness, metta and tonglen meditation

The roots of mindfulness meditation go back to Buddhism [14, 15]. However, in the Western world, the concept of mindfulness has increasingly been investigated and used as a secularized meditation practice, e.g., in the health and education system, since the 1970s [16]. The practice of mindfulness interrupts the everyday stream of thoughts and brings one into contact with oneself by directing the attention to the “here and now” [14, 17]. The aim is to cultivate an attitude that is non-judgmental but characterized by curiosity and acceptance [18, 19].

Directly connected with this mindful attitude is Metta, a characteristic attribute of Buddhism and meditation form of its own. Metta linguistically originates from Pali meaning “goodness of heart”, or “loving kindness” [20]. It is to be generated by the visualization of a power source of love in the heart area. Inwardly repeated “Metta sentences”, such as “may I and all beings be able to deal well with illness and pain”, aim to support the feeling of heart warmth, benevolence and compassion in the individual. Those feelings can be spread through visualization and lead to connectedness [20, 21].

A further development of Metta Meditation is Tonglen Meditation, which emphasises the compassionate interpersonal relationship (for review, see McKnight, 2012). As the central element is breathing, it is also called “heart breathing”. The exhalation corresponds to the emission of visualized heart warmth, benevolence and compassion (Metta). The inhalation corresponds to the absorption and resolution of negative aspects (e.g., pain) by the attentive and compassionate heart warmth [22, 23].

Research on Tonglen Meditation indicates improvement in the areas of self-compassion and general humanity [23]. Research on Metta/loving-kindness Meditation in neuroscientific studies suggest that the cultivation of positive emotions such as kindness and compassion influence the activation of neuronal circuits associated with empathy [24] and affective regulation (e.g., anxiety or mood) [25]. Various meta-analyses show that mindfulness-based interventions can relieve stress, anxiety, depression, burnout and pain [26–28]. In addition, studies support the use of mindfulness and compassion-based programs for staff involved in patient care [29–31]. Particularly in the field of oncology, a significant positive influence on life satisfaction and a reduction of compassion fatigue has been demonstrated [32]. In palliative care, research has shown that engagement with spirituality and meaning in Buddhist-leaning programs leads to improvements in the areas of compassion for the dying, self-confidence, job satisfaction and workload [12, 33, 34].

### Aims and contribution to the field

People who work in a palliative care context are faced with high demands, as they should neither be overwhelmed by existential needs, nor jaded and emotionally blunted due to chronic exposure. Rather, they should remain human, accessible and in contact with themselves. Nevertheless, interventions for palliative care providers addressing these attributes have hardly been researched. Studies in this area emphasize the need for corresponding techniques, especially those that are integrated into everyday working life [12, 13]. Orellana-Rios et al. (2018) were able to show that an “on the job” mindfulness and compassion program lasting several weeks in a palliative care centre, reduced the intensity of the perceived stress and strengthened self-care. Participants reported benefits in terms of emotion regulation and interpersonal skills, though there was no strengthening of general compassion. Orellana-Rios et al. were the first to find evidence that meditation improved knowledge or skills or changed the attitude of the participants [12]. Furthermore, there is a need for comparative data regarding the implementation of such programs at other locations. Since a comparable course was conducted and internally evaluated at our centre, we decided to retrospectively analyse these data scientifically. The main objective was to find out how such a training affects a multiprofessional team and the common work and whether it is feasible. In order to approach this, the existing data on quality assessment was used. For this purpose, the following research areas were examined: (1) Feasibility and acceptance of the course, (2) satisfaction and impact on the participating employees and (3) possibilities and limits in the mediation of concrete course contents regarding the cognitive and affective learning outcome as well as the development of a positive attitude.

## Methods

### Design

The present work is a retrospective descriptive study with self-assessment. For this purpose, a learning objectives survey and a qualitative analysis of open text responses were conducted. Data was collected immediately after completion of the course as a routine evaluation as part of the quality management of the specialized palliative care unit. This work has therefore been conducted using available quality assessment data.

The qualitative analysis makes it possible to learn about participants’ experiences from their personal perspective and to find out other information not included in the questionnaire. Qualitative analysis does not aim at representativeness, therefore a small number of participants may be sufficient. The aim is to obtain more detailed information about the personal experiences and to relate these to the quantitative evaluation in the present study. For transparency, a standard of quality of qualitative research, the steps of the analysis are shown under qualitative analysis.

### Measures

The evaluation was carried out in the form of a self-constructed questionnaire consisting of three parts:

- 1. Demographic and participation/program attendance data
- 2. Satisfaction data
- 3. Comparative Self-Assessment (CSA)

Part two and three are a self-assessment by the participants.

Part one comprised eight questions enquiring about demographic and participation data, as well as a free text field about previous experience with mindfulness.

Part two comprised three Likert-based questions concerning course satisfaction – 1) Please rate your level of overall satisfaction with the course. 2) Would you recommend the course to other employees working in palliative care? 3) Please rate your level of overall satisfaction with the instructor ‘s way of teaching. 4) Do you plan to practice the exercises that you learned in the course? – and a free text field to address experiences, feelings, peculiarities, criticism and wishes (see Table 5 for results).

Part three comprised 22 learning objectives that had to be self-assessed retrospectively before and after the course respectively (post-then). Parts of the learning objectives were based on the Freiburg Mindfulness Inventory [35, 36]. The other items were generated by group discussion and in consultation with the mindfulness and meditation teacher. The German and English versions of the questionnaire are available in Supplemental Information.

Part one of the questionnaire addresses the study objective "feasibility and acceptance of the course”. Part two addresses the study objective of “satisfaction”.

The third part of the questionnaire aims to investigate the impact of such training on the basis of learning gains. In this way, it can be determined whether the participants can learn something for themselves. The learning gain can also be used to address the third study question, that means topoint out the possibilities, but also the limits of the training, depending on how high or low the learning gain is.

### Participants

One course on “Mindfulness and Compassion” was offered to the multi-professional team members of the inpatient unit (mainly physicians, nurses, physiotherapists, psychologists) of a specialized palliative care unit at a University Hospital in Germany. Participation was voluntary and participants were recruited by invitation and internal advertising. Program attendance was not registered during the course. Team members of the liaison and volunteer service, as well as associated employees, e.g., from music therapy, pastoral care or social work were also invited, but couldn´t attend on a regular basis due to the structure of the course.

### Course

**Fig. 1:**
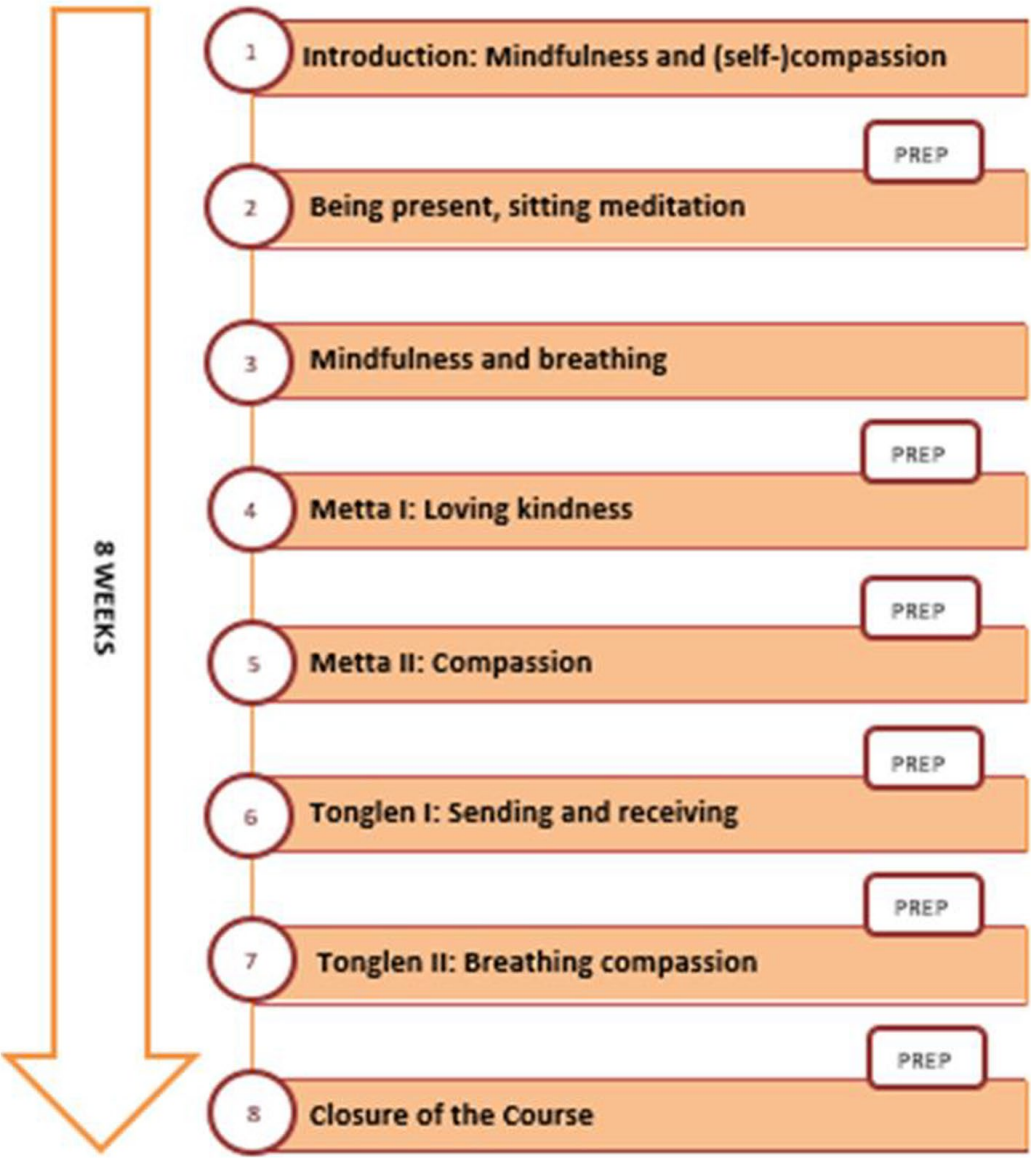
Structure of the course – Graphic representation of the structure of the course

For the course, a course outline, and a teacher that had already successfully run a course in another palliative care centre, were chosen [12]. The mindfulness and compassion course had a duration of eight weeks. The aims were to introduce the concept of mindfulness, to learn about and practice Metta/loving-kindness and Tonglen and to learn how to integrate techniques of self-care into everyday working life. It was designed and provided by a mindfulness/meditation teacher, former Buddhist monk and Tonglen expert. Figure 1 summarizes the structure and main topics of the course. At the beginning, an introductory full-day seminar was held as a kick-off. In the further course of eight weeks, the teacher was on site at the ward one day per week. On this day, he offered short meditations (5–15 min) every hour as well as individual counselling breaks on request. Additionally, a two hour seminar took place on six evenings over the course of eight weeks, in which theoretical input was imparted, different techniques were introduced and there was an opportunity for reflection and exchange. Generally, techniques and attitude changes were also to be gradually implemented into the personal (work) routine. To familiarize, practice and consolidate a variety of techniques, different practice elements were offered (see Table 1). These were to be deepened in the various events or the participants could turn to the trainer with uncertainties and questions. The course was held as part of advanced training, therefore the participation was free of charge and calculated as working time. The costs were covered by the centre’s support association.

**Table 1:** Practice elements of the mindfulness and compassion course

Introductory Day (1)	Mindfulness-Days (7)	Prep. Evening Seminars (6)	Additional Practices
*Duration: 6 h*	*Duration: 8 h*	*Duration: 1.5 h*	
Project presentation	Hourly 5–15 min guided meditation (announcement: sounding singing bowl)	Theoretical input	Metta/Tonglen practice during patient care/contact with relatives
Background information	One-to-one sessions or guidance if needed	Introduction of new techniques	Mindful breaks
Basic techniques of mindfulness meditation (e.g. sitting, walking and breathing meditation)	“by the way” practices (see additional practices)	Practice time	Practice anchors during daily work routines
Self-care and compassion		Reflection and exchange	Meditation at home with audio CD

### Data collection

Evaluation questionnaires were distributed in paper form as part of the closure session of the course. The questionnaire can be found under supplementary files (Mindfulness questionnaire). Participation was open to every employee who had attended the course in full or in part. Conducting the survey took place as part of a routine quality assurance survey. Therefore, all course participants were included and a high response rate could be expected even if participation was voluntary. The sample corresponds to the description of the participants. Additional questionnaires were accessible for later participation and further participants respectively. As in every regular course evaluation, participation was voluntary and anonymous. In a timeframe of two weeks, the evaluation questionnaires were collected in a box ensuring that no tracing of the participants was possible. With their submission, the participants gave their consent to the evaluation. For the retrospective scientific analysis of the routine evaluation, a positive vote of the appropriate ethics committee is available.

### Statistical analysis

Quantitative data was analysed using Microsoft Excel version 2205 (Microsoft, Redmond, WA, U. S. A.) and IBM SPSS Statistics version 28.0.1.1 (IBM, Armonk, NY, U. S. A). For Questionnaire part one (demographic data and participation) and part two (satisfaction), descriptive analyses were conducted to obtain means/medians, range, standard deviations and percentages. Satisfaction was asked about in an understandable way, by allowing the participants to rate and indicate the questions on satisfaction with the course and the trainer himself on a five-point scale from very satisfied to very dissatisfied.

Questionnaire part three measured the learning gain of the participants in a post-then procedure. In 22 items, different aspects of the course content were given. These learning objectives were developed collaboratively within the research group, but primarily with the instructor. He could specifically state what he wants to and can achieve with the course. In addition, the literature on courses that have already been carried out and an existing mindfulness inventory were used as a guide [12, 35, 36]. The participants rated these items after the course for two time points: First, they were asked to look back and assess their own skills before the course and, second, to assess their skills after the course. This type of learning objectives survey reduces the response shift bias [37], because the participants can better assess their own abilities based on the knowledge they initially got through the course. The answers were coded according to a Likert scale from one (best) to five (worst). For a few items, some participants chose values in the middle of two points. The average of these points was used in these cases (e.g., 2.5 for a rating in the middle of 2 and 3).

The post-then procedure allows the calculation of the Comparative Self-Assessment (CSA) Gain according to Raupach et al. [38]. For the CSA-Gain calculation, the learning objectives of a course are transformed into a questionnaire. Thus, the questionnaires used for CSA Gain analysis do not serve as psychometric tests but reflect the learning objectives directly. Therefore, a CSA Gain analysis addresses the level of learning objectives achieved in a course rather than psychometric changes. For this reason, the questionnaire is not validated but the method to measure CSA Gain analysis. High Pearson’s *r* correlations were found when compared to a prospective performance measurement (*r* = 0.975, Ref. 37) or overall ratings of a course (*r* = 0.94, Ref. 38). A prospective, longitudinal intervention study with *n* = 636 students provided validity evidence [39].

The CSA Gain method, used as a form of outcome measurement, identifies changes in relation to the objectives under study. Compared to other learning outcome measures that only test knowledge at a specific point in time, the calculation of Comparative Self-Assessment includes information about the participants’ initial level of performance. It therefore provides information about the growth in knowledge, skills and attitudes acquired over the course duration. Furthermore, comparative self-assessments have a high validity compared to isolated self-assessments [38]. The reason for this is that the self-assessment ability of a given person is relatively stable over time. The decisive factor for validity is of course the quality of the statements/learning objectives used for the self-assessment [38].

The formula originally published by Raupach et al. (2012) reads CSA Gain [%] = (mean pre – mean post) / (mean pre – 1) × 100. The CSA Gain gives the percentage change in the level of knowledge, skill or attitude in values ranging from -100% to + 100%. To calculate 95% confidence intervals and standard error of the CSA Gain, we calculated the individual learning gain values (ILG) first, according to Ref. 39. To this end, we used the formulas (1) ILG = 0 for pre = post, (2) ILG = (pre – post) / (pre – 1) × 100 for pre > post and (3) ILG = (pre – post) / (post – 1) × 100 for pre < post. Here, *pre* < *post* translates into a deterioration of the individual learning gain reported. The use of the third formula accommodates for these cases, which occurred rarely in our study.

With these formulas, CSA Gain equals the mean of ILG and gives the same result as the original formula [38] in a usual teaching situation where most participants improve their knowledge, skills or attitude and no participant reports a deterioration. Participants who failed to rate individual items for the post or then time points were excluded from the calculation of this item. The values of CSA Gain, 95% confidence interval and standard error, are given in percent and rounded to one decimal place (Table 7).

### Qualitative analysis

One of the authors, an academic nurse with experience in qualitative research, who is currently active in palliative care education, carried out the qualitative analysis. The qualitative analysis was executed independently of the quantitative analysis.

**Fig. 2:**
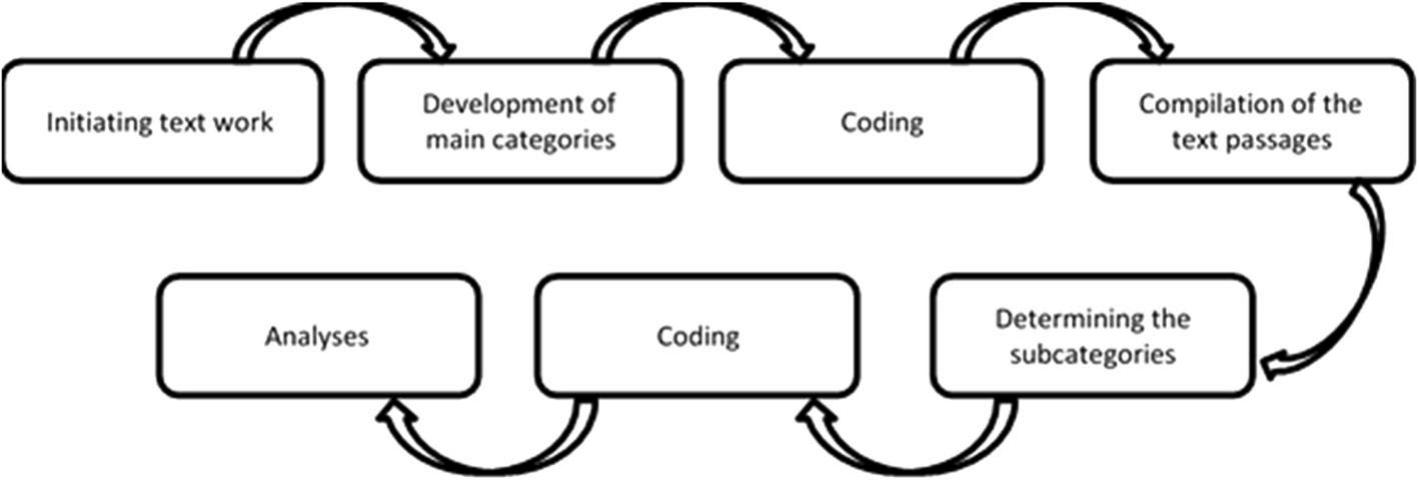
Steps of the structuring content analysis of Kuckartz

The answers to the free text field “experiences, feelings, peculiarities, criticism and wishes” were evaluated by structuring qualitative content analysis according to Kuckartz (2016), first by the author mentioned above and then, for quality assurance purposes, by a second author, as described below. The categories of the main and subcategories were formed inductively in a multi-stage process on the material, whereby first it was roughly encoded along main categories. The next step was to further develop and differentiate the categories in the material. After the entire data material had been coded a second time, the category-based evaluation followed [40]. The exact sequence of the evaluation is shown in Fig. 2. The analysis was based on the main and subcategories, as a complex analysis of the interrelations was not possible due to the small amount of data.

After analysis, a second author, a physician and mindfulness coach, also independently categorized the responses. No differences worth discussing emerged in the analysis. The results were presented to the research group and accepted by all of them. The free text field “Previous experience with mindfulness” was summarized and displayed as a list.

## Results

### Demographic data

Of the 27 team members working in the palliative care centre at that time, 25 participated in the course and 24 rated it based on the evaluation (96%). Half of the participants were nurses, representing the staff majority of the specialized palliative care team. Table 2 shows the demographic characteristics.

**Table 2:** Demographic data of the participants (*n* = 24)

Profession (frequencies)	
Nurses	12 (50%)
Physicians	6 (25%)
Physiotherapists	2 (8%)
Psycho-oncologists	2 (8%)
Massage Therapists	1 (4%)
Administration assistant	1 (4%)
**Median age (range)**	39.5 (26–62) years
**Gender**	
Female	20 (83%)
Male	4 (17%)

### Previous experience with mindfulness

The evaluation shows that 66% of the participants already had previous experience in the field of meditation and/or mindfulness. The corresponding free text field answers provide an insight into the type of experience (see Table 3).

**Table 3:** Previous experience with mindfulness

**No experience**	10 (42%)	
**Previous experience**	14 (58%)	Free text field answers: Walking meditation, Shi-Bashi, Qui-Gong, fragrance meditation Autogenic training Everything I do, I do consciously (I try to) Literature “The Little Everyday Buddhist” Years of meditation experience Global issue of mindfulness, with patients Short meditation sessions in one holiday Guided meditations (audio CD) As part of my psychomotor training 7 (8) week training after J. Kabat-Zinn and 2 × 5 days meditation and mindfulness training Progressive muscle relaxation 2 weeks evening group mindfulness Breathing exercises, meditation

### Participation

72% of the team members took part in the introductory seminar day on a Saturday. 58% took part in more than half of the mindfulness days during work. Still, 48% joined at least 4 of the 6 preparatory evening events during free time (see Table 4).

**Table 4:** Compliance (Participation Data)

Participation	value
Participation on the introductory seminar day (Saturday)	18/25 (72%)
Participation in at least 4 of the 7 Mindfulness-Days	14/24 (58%)
Median (range) of attended Mindfulness-Days (7)	4 (0–7)
Participation on at least 4 of the 6 prep. evening seminars	12/25 (48%)
Median (range) of attended prep. evening seminars (6)	3 (0–5)

### Satisfaction data

91% of the respondents showed moderate to high satisfaction with the course and the teacher (Likert Scale 1–2) and would recommend it for palliative care. See Table 5 for means and corresponding standard deviations. Regarding further implementation, 83% declared they wanted to use the techniques in the future.

**Table 5:** Satisfaction data

VARIABLE^1^	MEAN (SD)
Satisfaction with the Course	2.21 (1.22)
Recommendation for palliative care	1.50 (0.70)
Satisfaction with meditation teacher	1.75 (0.78)

^1^) Likert scale: 1 (very satisfied/positive) to 5 (very dissatisfied/negative)

### Qualitative data

18 of 24 participants commented in the free text field “experiences, feelings, peculiarities, criticism and wishes”, of which only one contained a negative evaluation, which referred to personal feelings. From the detailed analysis, three main categories emerged, providing feedback about the trainer and the techniques in (1) “the course”, as well as about the implementation and effects regarding (2) “professional life” and (3) “personal life” respectively. A fourth category summarizes statements about “empathy”. Table 6 contains corresponding examples for the categories.

1. The Course

**Table 6:** The code-system

Category	Codes	Examples
**The Course**	Trainer	*“the benevolent charisma of the mentor”* *“I personally didn't like the way the instructions were given”*
	Techniques	*“Variety of applications of mindfulness”* *“I like the idea/concept of the course”*
**Professional Life**	Implementation	*“The implementation into the daily routine of the hospital through short practice units is possible”*
	Effects	*“Perhaps the participants are now more mindful of themselves but not of their colleagues.”* *“to do something for yourself during working hours”*
**Personal Life**	Implementation	*“Variety of uses for mindfulness in everyday life”* *“the journey through the body”* *“the benevolent heart”*
	Effects	*“positive mood changes after the exercises”* *“In one of the mindfulness days I came with physical complaints (neck) that had completely disappeared after the meditation!”*
**Empathy**		*“Feeling empathy for my partner, my counterpart”* *“Empathy—*> *Compassion”*

This category summarizes statements referring to “the trainer” and “the techniques”. The respondents were split regarding the trainer. While most participants liked his benevolent and calm nature, he was too calm, too familiar and not formal enough for others. The variety of techniques and the concept were generally perceived as good, with the “Tonglen Meditation” and the “Four Steps of the Separation of Body and Mind” being emphasized.

(2) Professional Life

This category summarizes statements referring to “the implementation” and “the effects” of the learned techniques regarding everyday working life. The participants reported that in the form of short practice units, application of the techniques is possible and some improvements in mood were noticed.

Even though participants described that they need more routine in order to implement what they have learnt in everyday working life, the possibility to do something for themselves during work time was emphasized. However, not all participants feel that the training has increased their awareness of other team members.

(3) Personal Life

This category summarizes statements referring to “the implementation” and “the effects” of the learned techniques regarding personal life. Some participants found some parts of the training useful for themselves and were able to continue to put it into practice in their private lives. They saw the possibility of the private implementation in a variety of applications, whereby they recognized an influence on different levels; physical as well as psychological. In addition to positive mood changes, some participants described a reduction of physical complaints after a session. Participants with previous experience of doing mindfulness exercises were able to strengthen their application.

(4) Empathy

Empathy is the only category that could not be further subdivided. There are only a few statements that contain the words ‘compassion’ and ‘empathy’. The participants did not give any further explanations, so their statements could not be sorted into the other categories. All in all, the participants communicated feelings of strengthened empathy.

### CSA-Gain

**Fig. 3:**
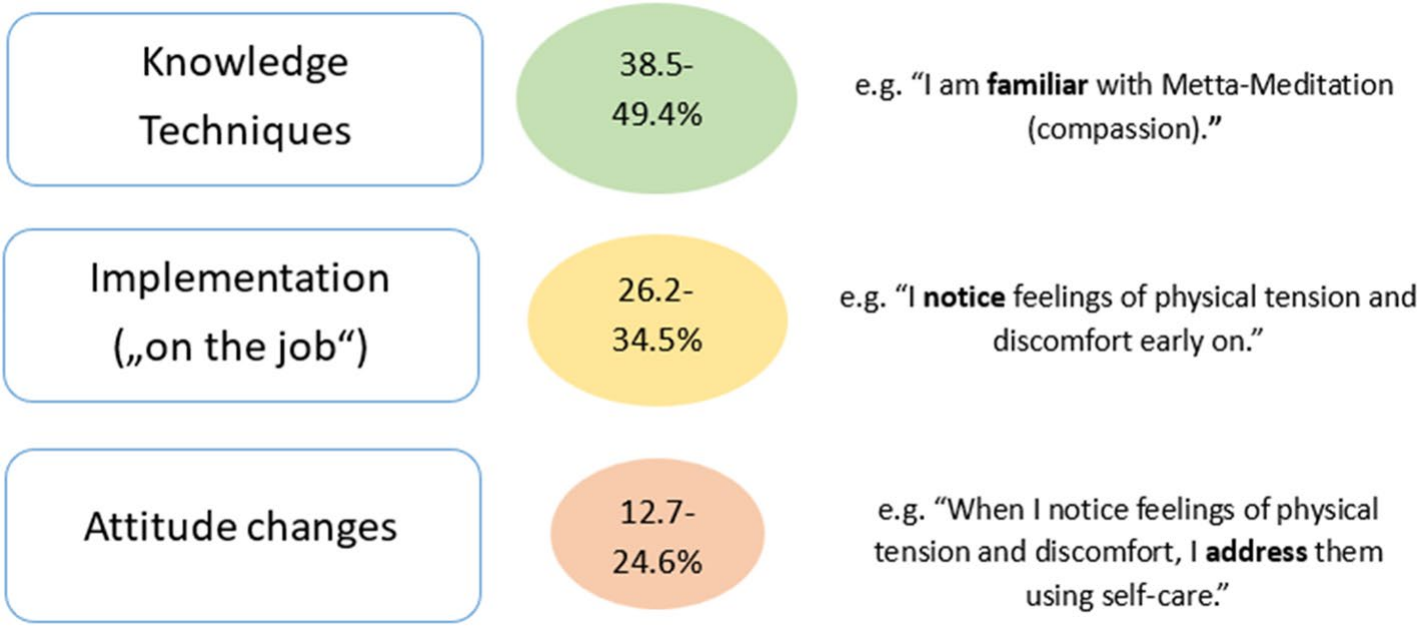
Summarized CSA-Gains for the different categories

**Fig. 4:**
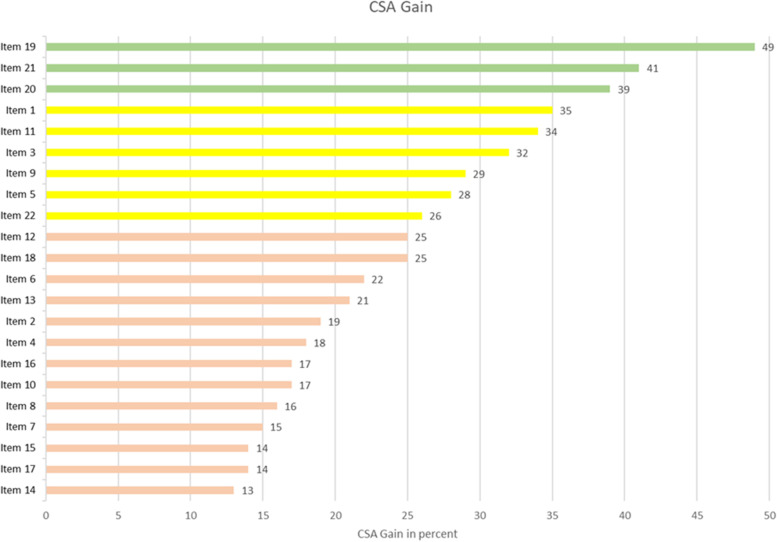
CSA Gain of each item

The learning increase (CSA-Gain) was high (38.5–49.4%) concerning knowledge and techniques. Items referring to the concrete implementation of learned skills in the work environment and teamwork (Items 7, 10 and 16), showed a low (15.1–16.9%) learning increase. A rather low (12.7–24.6%) increase was established concerning changes to attitude and deepened self-reflection (distancing from one’s own feelings, positive perception of the self). A moderate increase (26.2–34.5%) was found with the implementation of parts of the course content. Table 7 shows the CSA Gain for all items in ascending order and Fig. 3 summarizes them. Looking at Items 17 and 5, it becomes clear that the perception of one’s own feelings (Item 5) is improved, but there is no significant increase in the level of protective distancing from such feelings (Item 17). Figure 4 shows the items and their CSA gains in ascending order.

**Table 7:** Comparative Self-Assessment-Gain of all Items^a^

No	Item	*n*	CSAGain (%)	95% CI	SE (%)
Changes to Attitude					
14	I can see my mistakes and difficulties without judging myself for them	21	**12.7**	-2.3–27.7 **(n.s.)**	7.2
17	I can perceive my feelings without losing myself in them or suffering from them	21	**13.9**	-5.9–33.6 **(n.s.)**	9.5
15	I am patient with myself	21	**14.2**	2.0–26.4	5.8
7^a^	I experience a mindful contact with the other team members	21	**15.1**	-0.1–30.2 **(n.s.)**	7.3
8	I take time to eat (eat mindfully)	21	**16.3**	3.1–29.5	6.3
10^a^	Even in difficult situations I can adopt a compassionate attitude towards myself and others	21	**16.7**	1.7–31.6	7.2
16^a^	I am patient with others	21	**16.9**	-2.9–36.7 **(n.s.)**	9.5
4	Even in stressful or strained situations, I maintain contact with a kind of inner peace within myself	21	**17.5**	1.5–33.4	7.6
2	I use mindfulness/self-care techniques at home in everyday life	21	**18.7**	1.3–36.0	8.3
13	I can value myself	20	**20.8**	2.8–38.9	8.6
6	When I notice feelings of physical tension and discomfort, I address them in terms of self-care	21	**21.5**	4.9–38.1	8.0
18	During my work I feel connected to the “here and now”	21	**24.6**	2.5–46.7	10.6
12	When I am stressed or tense, I can consciously relax	21	**24.6**	8.9–40.3	7.5
Implementation					
22	I can use Tonglen to make stressful situations more bearable	21	**26.2**	11.6–40.8	7.0
5	I notice feelings of physical tension and discomfort early on	20	**27.5**	10.3–44.7	8.2
9	I have an “anchor” that grounds me and brings me into the “here and now”, e.g. between two patient conversations	21	**28.6**	10.9–46.3	8.5
3	I integrate mindfulness/self-care techniques into my professional everyday life on the palliative care ward	21	**31.7**	14.5–49.0	8.3
11	By using mindfulness techniques, I can gather strength during a working day	21	**34.1**	18.2–50.1	7.6
1	I am familiar with mindfulness/self-care techniques	21	**34.5**	12.4–56.7	10.6
Knowledge and Techniques					
20	I can use Metta meditation (compassion) to experience stressful situations more bearably	21	**38.5**	22.6–54.4	7.6
21	I am familiar with Tonglen (give and take meditation)	20	**41.3**	22.9–59.6	8.8
19	I am familiar with Metta meditation (compassion)	21	**49.4**	30.3–68.5	9.1

*Abbreviations CI* Confidence interval, *CSA* Comparative self-assessment, *n* Number of respondents, *n. s* Not significant, *SE* Standard error of the mean (i. e., of the CSA Gain)

1) The colours code for high (green), moderate (yellow) and low (red) learning increase

^a^) Items 7, 10 and 16 refer to social competence

## Discussion

The purpose of this project was to examine the feasibility and acceptance of a mindfulness and compassion course, as well as satisfaction with and influence of the course. In addition, the extent to which concrete course contents could be conveyed was also examined.

### Feasibility and acceptance

The mindfulness and compassion course, was successfully conducted in a specialized multi-professional inpatient palliative care team. The data show that the use of mindfulness practices such as mindfulness pauses can improve one’s perception of oneself or one’s surroundings. The participants indicate that they recognized the usefulness of the course in their daily work. It is notable that there are no negative comments in the free text answers, except for one personal feedback that refers to the teaching. Here, it can only be assumed, whether gratitude for such an offer that predominates and therefore there is no negative criticism because it was not perceived as such. However, it is also possible that only those who perceived it as valuable and positive took the opportunity to report this via the free text field. Persons who found it negative may not have taken the time to indicate this in writing as well.

The acceptance of the course by participants is also due to its implementation during working hours [41]. Remembering to take time out for a break during the stressful daily routine on the ward is not always easy. Through the hourly active reminder given by the trainer, employees can become aware of the need for a short time-out. Allocating someone within the team to remind employees to take short, regular breaks should be considered. An occasional conversation can certainly bring about a short time-out; moreover, the use of mindfulness exercises can strengthen the effect of stress reduction.

### Satisfaction and influence

The course satisfaction rating shows a high overall satisfaction with the course and with the trainer. Furthermore, there is a strong desire to use these techniques in the future.

The divergent evaluation of the trainer may illustrate people’s individuality. However, it may also raise the question of whether the course was well suited to the target group. After all the choice was based on the experience of other palliative care centres [12], because otherwise there was a lack of experience for the targeted selection. Nevertheless, the evaluation of the course concept was positive, so that the way of using the techniques seems appropriate. Team members highlighted different aspects in their statements, but overall, the knowledge and skills acquired seem to have led to an increased feeling of being able to take care of themselves during working hours. In this context, short practice units and short breaks were mentioned in particular. However, the qualitative analysis shows that more routine with the practices is needed for the team members to integrate and implement them in their everyday lives. Compassion training can have an impact on practice if it becomes a key component at different levels [42]. In the literature, such training is also seen as a way to reduce stress. It can promote emotional balance and recognition of emotions, as well as reduce emotional exhaustion [43]. However, the short-term focus of the training sessions can make it difficult for them to become permanent [12].

### Teaching concrete course content

In relation to learning gains, the application of the CSA Gain shows that the learning objectives that related to knowledge of techniques achieved the highest gains. This was followed by objectives relating to the concrete implementation of the learned skills in the work environment and in teamwork. The analysis was concluded with goals related to changes in attitude and deeper self-reflection. At the knowledge level, participants are usually most likely to be reached by courses. This is also the case here.

In order to take a closer look at the CSA Gain results, the proposed classification based on the CSA Gain was related to an existing category system, the German Qualifications Framework for Lifelong Learning (DQR) [44]. It is divided into “professional competence” and “personal competence”. “Professional competence” consists of the categories “knowledge” and “skills”, “personal competence” consists of the categories “social competence” and “independence”. In Table 8, the individual items are assigned to the categories and the average of the respective CSA Gains is shown.

**Table 8:** Classification of the items according to the categories of the German qualification framework for lifelong learning

*Competency*	Category	Items	Mean CSA Gain (%)
*Professional Competence*	Knowledge	19, 20, 21	43.1
	Skills	1, 3, 5, 9, 11, 12, 13, 22	28.5
*Personal Competence*	Social Competence	7, 10, 16	16.2
	Independence^a^	2, 4, 6, 8, 13, 14, 15, 17, 18	15.4

^a^Independence means the ability and willingness to act independently and responsibly, to reflect on one*’*s own actions and those of others, and to develop one*’*s own ability to act

The classification confirms that the most significant learning gain is in the area of “knowledge” (i.e., the “result of learning and understanding”). The second highest rated items were classified in the category “skills”, which describes the practical application of the acquired knowledge. The three items relating to teamwork (Items 7, 10 and 16) were placed in the category “social competence” and show a lower mean learning gain. The category “independence” means the ability and willingness to act independently and responsibly, to reflect on one’s own actions and those of others and to develop one’s own ability to act. The items in this category had the lowest mean CSA Gain. In summary, according to this categorisation, the mindfulness and compassion course had a higher impact on the professional than on the personal competence of the participants. In teaching and training, it is repeatedly shown that knowledge and skills are easier to teach and train than attitude. Attitude is often shaped by personal mindsets, experiences and backgrounds and is therefore not easy to teach or train. Here, rather self-made practical experiences and applying the techniques in real everyday situations can be helpful [45, 46]. The high impact on professional competencies is also evident here, as was the goal of this course. Compassionate health practice can be successfully perpetuated, especially if it becomes part of everyday work life and a key component [42].

The qualitative analysis complements the course evaluation, learning gain and assignment to specific competences with personal impressions of the participants. This finding supports and explains the CSA Gain results, which indicate a high gain in knowledge (e.g., Item 19 with 49.4%), but a rather low gain in attitude change (e.g., Item 14 with 12.7%) and dealing with feelings of discomfort such as healthy distancing (e.g., Item 17 with 13.9%) or striving for self-care (e.g., Item 6 with 21.5%). The literature suggests that recognizing one’s own perceptions and emotional responses may be the first step in alleviating stress [47]. In order for this to be possible, knowledge is first a prerequisite, whereby handling and changes only happen subsequently.

In the qualitative analysis, most answers could be assigned to the category “private life”. At first glance, this does not seem to correspond with the quantitative results, which show the lowest learning gains in terms of personal competence. However, since this category contains statements on personal aspects in general, it also includes effects that take place during working life or are related to working life (as one participant put it: “The rest; the short opportunities to interrupt the hectic daily routine”).

In the literature, such training is indicated as helpful in reducing stress and promoting the sense of well-being [48]. In order for this to happen, the participants must learn enough in the training sessions to enable them to apply this in their further everyday work while also simultaneously continuing to train. A basis for this has been created here. The aspect that further offers of mindfulness training promote resources must not be disregarded. Feasibility includes a permanent continuity of such offers. If this is not possible, the feasibility is limited. An important aspect is therefore the independent continuation of the participants, also in their free time. In a professional context, continuity can be strengthened through joint events and collegial exchange.

The CoViD-19 pandemic has led to an increased workload for medical staff in many places [49]. Palliative Care teams were also affected. Research showed that staff who had a higher level of self-compassion were resilient. This level increased with experience [50]. The continuity of such mindfulness training is therefore also important in calm times in order to be able to use it in more stressful times.

### Limitations

Due to the general conditions, this study has some limitations that may influence the validity of the conclusions. These include the small number of participants, which is limited by the size of the team. This small number may affect the internal validity of the study. However, the CSA Gain is methodologically designed in such way that valid results can also be obtained here. The data from at least 20 participants is shown to provide valid results [38]. Also in qualitative research, a small number of participants can be sufficient. In addition, this was a retrospective analysis of routine data based exclusively on participant self-assessment. The survey instruments have not been validated, but were primarily used for internal evaluation for quality assurance purposes. The course itself was also individually adapted to the setting and does not correspond to empirically tested concepts. In addition, it was not possible to evaluate a control group, as the study was performed retrospectively using quality management data. Regarding the impact of the course, there may be a limitation due to the short, eight-week duration of the course. Statements about continuation are not possible on the basis of the study.

## Conclusions

Quantitative and qualitative results complement each other and point to a common answer to the preceding questions. An “on the job” program with mindfulness and compassion-oriented exercises was possible and was not only accepted by the members of the palliative care team, but also perceived as satisfactory. As a recommendation for practice, it can be deduced that such training can be perceived as beneficial for teamwork and one’s own attitudes. However, it should take place over a longer period of time or be reinstated after a break to make permanence more likely. The direct integration of the training into the daily work routine can be passed on as a recommendation, even if it means investing a considerable amount of time. In order to make the training useful for the palliative care team, the teacher should be familiar with the framework conditions and special features of everyday hospital life, as mentioned in the qualitative data. A further study should be carried out as a controlled trial to assess the long-term impact on joint teamwork. In such future studies, the Kirkpatrick model [51] could be applied, which evaluates courses at the four levels of response, learning, behaviour and outcomes. Appropriate learning objectives and assessment items could be pre-determined. The aim is to find out whether there is a change in behaviour and whether the course meets the predefined objectives.

## Supplementary Information

**Additional file 1.**

**Additional file 2.**

## Data Availability

The datasets used and analyzed during the current study are available from the corresponding author on reasonable request.

## Abbreviations

CSA: Comparative Self-Assessment
ILG: Individual learning gain
SD: Standard deviation
SE: Standard error
95% CI: 95% Confidence interval
