# Supplementary material for: Mindfulness and compassion training on daily work with patients and within the multiprofessional palliative care team: a retrospective self-assessment study
Source: BMC Palliat Care. 2023 Apr 10;22:37. doi: 10.1186/s12904-023-01158-9 (PMC10084609; doi:10.1186/s12904-023-01158-9)
Supplement: Supplementary file 1 — Additional file 1. [file 12904_2023_1158_MOESM1_ESM.pdf]

Part 1 of 4

Did you attend the introduction on Saturday Jan., 6th 2018?

Yes ☐ No ☐

Did you attend at least 4 of the evening sessions on Tuesdays?

Yes ☐ No ☐

How many of the 6 evening sessions on Tuesdays did you attend? ______

Did you attend at least 4 of the mindfulness days on Wednesdays?

Yes ☐ No ☐

How many of the 6 mindfulness days on Wednesdays did you attend? ______

Age: _____years

Sex:

Female ☐ Male ☐

Profession

☐ Nurse ☐ Physician ☐ Physiotherapist ☐ Psychologist ☐ Social worker ☐ Other

Have you practiced mindfulness exercises before?

Yes ☐ No ☐

If yes, please specify: ____

Part 2 of 4

1. Please rate your level of overall satisfaction with the course.

very dissatisfied	rather dissatisfied	undecided	rather satisfied	very satisfied
☐	☐	☐	☐	☐

2. Would you recommend the course to other colleagues working in palliative care?

no	more likely no	maybe	more likely yes	yes
☐	☐	☐	☐	☐

3. Please rate your level of overall satisfaction with the instructor‘s way of teaching.

very dissatisfied	rather dissatisfied	undecided	rather satisfied	very satisfied
☐	☐	☐	☐	☐

4. Do you plan to practice the exercises that you learned in the course?

Yes ☐ No ☐

5. What are your most memorable moments of the course?

Experiences, feelings, peculiarities, criticism, wishes:

____

____

____

____

____

____

Part 3 of 4

How do you personally rate yourself regarding the following statements?

→ Note: BEFORE the course ←

No.	Item	strongly disagree	disagree	undecided	agree	strongly agree
1	I am familiar with mindfulness/self-care techniques.					
2	I use mindfulness/self-care techniques at home in everyday life.					
3	I integrate mindfulness/self-care techniques into my professional everyday life on the palliative care ward.					
4	Even in stressful or strained situations, I maintain contact with a kind of inner peace within myself.					
5	I notice feelings of physical tension and discomfort early on.					
6	When I notice feelings of physical tension and discomfort, I address them in terms of self-care.					
7	I experience a mindful contact with the other team members.					
8	I take time to eat (eat mindfully).					
9	I have an “anchor” that grounds me and brings me into the “here and now”, e.g. between two patient conversations.					
10	Even in difficult situations I can adopt a compassionate attitude towards myself and others.					
11	By using mindfulness techniques, I can gather strength during a working day.					
12	When I am stressed or tense, I can consciously relax.					
13	I can value myself.					
14	I can see my mistakes and difficulties without judging myself for them.					
15	I am patient with myself.					
16	I am patient with others.					
17	I can perceive my feelings without losing myself in them or suffering from them.					
18	During my work I feel connected to the “here and now”.					
19	I am familiar with Metta meditation (compassion).					
20	I can use Metta meditation (compassion) to experience stressful situations more bearably.					
21	I am familiar with Tonglen (give and take meditation).					
22	I can use Tonglen to make stressful situations more bearable.					

Part 4 of 4

How do you personally rate yourself regarding the following statements?

→ Note, AFTER the course, i. e., now ←

No.	Item	strongly disagree	disagree	undecided	agree	strongly agree
1	I am familiar with mindfulness/self-care techniques.					
2	I use mindfulness/self-care techniques at home in everyday life.					
3	I integrate mindfulness/self-care techniques into my professional everyday life on the palliative care ward.					
4	Even in stressful or strained situations, I maintain contact with a kind of inner peace within myself.					
5	I notice feelings of physical tension and discomfort early on.					
6	When I notice feelings of physical tension and discomfort, I address them in terms of self-care.					
7	I experience a mindful contact with the other team members.					
8	I take time to eat (eat mindfully).					
9	I have an “anchor” that grounds me and brings me into the “here and now”, e.g. between two patient conversations.					
10	Even in difficult situations I can adopt a compassionate attitude towards myself and others.					
11	By using mindfulness techniques, I can gather strength during a working day.					
12	When I am stressed or tense, I can consciously relax.					
13	I can value myself.					
14	I can see my mistakes and difficulties without judging myself for them.					
15	I am patient with myself.					
16	I am patient with others.					
17	I can perceive my feelings without losing myself in them or suffering from them.					
18	During my work I feel connected to the “here and now”.					
19	I am familiar with Metta meditation (compassion).					
20	I can use Metta meditation (compassion) to experience stressful situations more bearably.					
21	I am familiar with Tonglen (give and take meditation).					
22	I can use Tonglen to make stressful situations more bearable.					
