# Supplementary material for: Mindfulness and compassion training on daily work with patients and within the multiprofessional palliative care team: a retrospective self-assessment study
Source: BMC Palliat Care. 2023 Apr 10;22:37. doi: 10.1186/s12904-023-01158-9 (PMC10084609; doi:10.1186/s12904-023-01158-9)
Supplement: Supplementary file 2 — Additional file 2. [file 12904_2023_1158_MOESM2_ESM.pdf]

Teil 1 von 4:

Waren Sie beim Einführungs-Samstag am 06.01.2018?

ja ☐ nein ☐

Haben Sie vier oder mehr der Dienstag-Abend-Veranstaltungen besucht?

ja ☐ nein ☐

Wieviele, der 6 Dienstag-Abend-Veranstaltungen haben Sie besucht? _______

Haben Sie vier oder mehr der Mittwoch-Achtsamkeitstage miterlebt?

ja ☐ nein ☐

Wieviele der 7 Mittwoch-Achtsamkeitstage haben Sie miterlebt? _______

Alter: _________ Jahre

Geschlecht:

weiblich ☐ männlich ☐

Beruf:

Pflege	Arzt/Ärztin	Physiotherapie	Psychologie	Sozialdienst
☐	☐	☐	☐	☐

weitere: _____________

Vorerfahrung mit Achtsamkeitstechniken:

ja ☐ nein ☐

Wenn ja, welche: ____

Teil 2 von 4:

1. Wie zufrieden waren Sie insgesamt mit dem Kurs?

sehr unzufrieden	eher unzufrieden	teils/teils	eher zufrieden	sehr zufrieden
☐	☐	☐	☐	☐

2. Würden Sie den Kurs anderen MitarbeiterInnen im palliativen Bereich empfehlen?

nein	eher nicht	vielleicht	eher ja	ja
☐	☐	☐	☐	☐

3. Wie zufrieden waren Sie mit der Art der Kursleitung?

sehr unzufrieden	eher unzufrieden	teils/teils	eher zufrieden	sehr zufrieden
☐	☐	☐	☐	☐

4. Planen Sie, die im Kurs erlernten Techniken in Zukunft einzusetzen?

ja ☐ nein ☐

5. Was ist Ihnen in besonderer Erinnerung geblieben?

Erlebnisse/ Gefühle/ Besonderheiten/ Kritik/ Wünsche:

____

____

____

____

____

____

Teil 3 von 4:

Wie schätzen Sie sich persönlich bezüglich der folgenden Aussagen ein?

→ Achtung: Zum Zeitpunkt VOR dem Projekt ←

Nr.	Item	Trifft gar nicht zu	Trifft eher nicht zu	neutral	Trifft eher zu	Trifft voll zu
1	Ich bin vertraut mit Techniken der Achtsamkeit/ Selbstfürsorge.					
2	Ich wende Techniken der Achtsamkeit/ Selbstfürsorge zu Hause im Alltag an.					
3	Ich integriere Techniken der Achtsamkeit/ Selbstfürsorge in meinen beruflichen Alltag auf der Palliativstation.					
4	Auch in belastenden Situationen behalte ich Kontakt zu einer Art innerem Frieden in mir.					
5	Ich nehme Gefühle der körperlichen Anspannung und des Unbehagens frühzeitig wahr.					
6	Wenn ich oben genannte Gefühle wahrnehme, gehe ich diesen im Sinne der Selbstfürsorge nach.					
7	Ich erlebe einen achtsamen Kontakt zu den anderen Team-Mitgliedern.					
8	Ich nehme mir Zeit zum Essen (esse achtsam).					
9	Ich habe einen „Anker", der mich z. B. zwischen zwei Patientengesprächen ins „Hier und Jetzt" bringt.					
10	Auch in schwierigen Situationen kann ich eine mitfühlende Haltung einnehmen.					
11	Durch die Anwendung von Achtsamkeitstechniken kann ich während eines Arbeitstags Kraft sammeln.					
12	Wenn ich angespannt bin, kann ich mich bewusst entspannen.					
13	Ich kann mich selbst wertschätzen.					
14	Ich kann meine Fehler sehen, ohne mich selbst dafür zu verurteilen.					
15	Ich bin geduldig mit mir selbst.					
16	Ich bin geduldig mit anderen.					
17	Ich kann meine Gefühle wahrnehmen, ohne mich darin zu verlieren oder darunter zu leiden.					
18	Während meiner Arbeit fühle ich mich mit dem „Hier und Jetzt" verbunden.					
19	Ich bin vertraut mit Metta-Meditation (Mitgefühl).					
20	Ich kann Metta-Meditation (Mitgefühl) anwenden, um belastende Situationen erträglicher zu erleben.					
21	Ich bin vertraut mit Tonglen (Geben und Nehmen-Meditation).					
22	Ich kann Tonglen anwenden, um belastende Situationen erträglicher zu erleben.					

Teil 4 von 4:

Wie schätzen Sie sich persönlich bezüglich der folgenden Aussagen ein?

→ JETZT, zum Zeitpunkt NACH dem Projekt ←

Nr.	Item	Trifft gar nicht zu	Trifft eher nicht zu	neutral	Trifft eher zu	Trifft voll zu
1	Ich bin vertraut mit Techniken der Achtsamkeit/ Selbstfürsorge.					
2	Ich wende Techniken der Achtsamkeit/ Selbstfürsorge zu Hause im Alltag an.					
3	Ich integriere Techniken der Achtsamkeit/ Selbstfürsorge in meinen beruflichen Alltag auf der Palliativstation.					
4	Auch in belastenden Situationen behalte ich Kontakt zu einer Art innerem Frieden in mir.					
5	Ich nehme Gefühle der körperlichen Anspannung und des Unbehagens frühzeitig wahr.					
6	Wenn ich oben genannte Gefühle wahrnehme, gehe ich diesen im Sinne der Selbstfürsorge nach.					
7	Ich erlebe einen achtsamen Kontakt zu den anderen Team-Mitgliedern.					
8	Ich nehme mir Zeit zum Essen (esse achtsam).					
9	Ich habe einen „Anker", der mich z. B. zwischen zwei Patientengesprächen ins „Hier und Jetzt" bringt.					
10	Auch in schwierigen Situationen kann ich eine mitfühlende Haltung einnehmen.					
11	Durch die Anwendung von Achtsamkeitstechniken kann ich während eines Arbeitstags Kraft sammeln.					
12	Wenn ich angespannt bin, kann ich mich bewusst entspannen.					
13	Ich kann mich selbst wertschätzen.					
14	Ich kann meine Fehler sehen, ohne mich selbst dafür zu verurteilen.					
15	Ich bin geduldig mit mir selbst.					
16	Ich bin geduldig mit anderen.					
17	Ich kann meine Gefühle wahrnehmen, ohne mich darin zu verlieren oder darunter zu leiden.					
18	Während meiner Arbeit fühle ich mich mit dem „Hier und Jetzt" verbunden.					
19	Ich bin vertraut mit Metta-Meditation (Mitgefühl).					
20	Ich kann Metta-Meditation (Mitgefühl) anwenden, um belastende Situationen erträglicher zu erleben.					
21	Ich bin vertraut mit Tonglen (Geben und Nehmen-Meditation).					
22	Ich kann Tonglen anwenden, um belastende Situationen erträglicher zu erleben.					
